# The Bill Approved by the College Council

**Published:** 1916-08-05

**Authors:** 


### NURSES' REGISTRATION, 1916.

### The Bill Approved by the College Council.

The following is the fourth Draft, as approved by the Council of the College of Nursing, Ltd., July 27, 1916, of a Bill (6 and 7 Geo. 5) to provide for the registration of nurses:—

Be it enacted by the King's most Excellent Majesty, by and with the advice and consent of the Lords Spiritual and Temporal, and Commons, in this present Parliament assembled, and by the authority of the same as follows:—

1. Short Title.—This Act may for all purposes be cited as the Nurses' Registration Act, 1916.

2. The College of Nursing.—(1) The name of the College of Nursing, Limited, shall be changed to "the College of Nursing," and it shall be entitled to bear that title without the addition of the word "Limited."

The General Nursing Council.—(2) The College of Nursing shall for the purposes of this Act act by the Council thereof (which shall be entitled "The General Nursing Council" and is hereinafter called "the Council") as regulated by this Act and by Rules made thereunder.

(3) Every nurse registered under this Act shall be entitled to a vote at elections of the Council, and, if registered on the General Register, without further fee to become a member of the College of Nursing.

3. Register of Nurses.—It shall be the duty of the College of Nursing to form and to keep a General Register of Nurses, a Supplementary Register of Male Nurses, and a Supplementary Register of Mental Nurses, and each such Register is hereinafter in this Act included in the term "the Register." The Register already formed by the College of Nursing, Limited, shall be the first Register under this Act.

4. Rules.—(1) Rules shall be made under this Act—

(i) regulating the constitution and proceedings and the mode of election and of retirement of the Council and providing for the representation thereon of the Privy Council and any Government Department, of the Nurse Training Schools, of the medical profession, and of the nurses on the Supplementary Registers: provided that

(*a*) not less than two-thirds of the Council shall be elected by the nurses on the General Register under this Act;

(*b*) the election of representatives of the nurses on the General Register and on any Supplementary Register shall be by voting papers to be transmitted through the post in the prescribed manner, and each person voting shall be entitled to give one vote for each of any number of candidates not exceeding the number of members of the Council to be elected to represent the nurses on the General Register or on a Supplementary Register as the case may be;

(*c*) the first Council to be elected under the rules shall come into office on the expiration of a period of two years after the passing of this Act;

(ii) regulating the issue and cancellation of certificates of registration and the conditions of admission to the Register;

(iii) regulating the course of training and the examination of nurses intending to be registered and the appointment of examiners;

(iv) regulating the admission to the Register of persons already in practice as trained nurses at the passing of this Act;

(v) regulating the admission to the Register of persons registered in any British Possession in which a Nurses' Registration Act is in force subject to such conditions and qualifications as the rules may prescribe;

(vi) providing for the publication of the Register with such particulars as the rules may prescribe;

(vii) providing for the temporary or permanent removal from the Register by the Council of any nurse for such causes or offences and after such inquiry as may be prescribed but subject to the appeal provided for by this Act;

(viii) regulating the procedure for the restoration to the Register of any nurse so removed;

(ix) providing for the establishment of and for regulating the powers of Local Boards for any parts of the United Kingdom for the purposes of this Act; and

(x) otherwise for the purposes of this Act.

(2) Rules under this Act shall be made by the Council, but shall have no effect until they have been approved by the Privy Council, and the Privy Council may approve the rules subject to such modification as the Privy Council think proper.

5. Provision as to the First Council.—(1) On the passing of this Act and for a term of two years the Council shall consist of the following forty-five persons so long as they may be willing to act:—

[Space left here for names to follow.]

(2) Casual vacancies may be filled up by co-optation.

6. Provision for Existing Nurses.—Any person who within three years from the passing of this Act claims to be registered thereunder shall be so registered, provided such person is at least twenty-one years of age and is of good character, and is qualified for registration under the conditions laid down by the College of Nursing, Limited, until and unless modified by rules made under this Act.

7. Who may be Registered.—At the expiration of the said term of three years any person who claims to be placed on the General Register under this Act shall be entitled to be so registered, provided that such person is at least twenty-one years of age and is of good character, and has had the training under a definite curriculum prescribed by the Council in a Nurse Training School or Schools recognised by the Council. Any nurse whose name is placed on the General Register and who holds a certificate of the Fever Nurses Association, or its equivalent, granted under conditions approved by the Council, shall be entitled on payment of a registration fee to have the words "also trained in fever nursing" added to her record in the Register.

8. A male nurse may claim to be placed upon the Supplementary Register of Male Nurses provided that such person has had the training under a definite curriculum prescribed by the Council in a Nurse Training School or Schools recognised by the Council, or holds a certificate of similar training as a nurse authorised by the Lords Commissioners of the Admiralty for the sick berth staff of the Royal Navy, or as a nurse authorised by the Army Council for soldiers of the Royal Army Medical Corps, on satisfying the Council that he is twenty-one years of age and of good character.

9. An asylum-trained nurse, who holds a certificate of the Medico-Psychological Association, or its equivalent, granted under conditions approved by the Council, or who has qualified as a mental attendant in the Royal Army Medical Corps, may claim to be placed upon the Supplementary Register of Mental Nurses on satisfying the Council that he or she is twenty-one years of age and of good character.

10. Fees.—Every candidate for examination or registration shall pay to the College of Nursing such fee as may be prescribed by the Rules.

11. Penalty for Pretending to be Registered.—From and after the publication of the first Register no person shall be entitled to take or use the name or title of registered nurse, or of registered male nurse or of registered mental nurse (either alone or in combination with any other word or words or letters), or any name, title, addition, or description implying that he or she is registered under this Act, or is recognised by law as a registered nurse, or as a registered male nurse, or as a registered mental nurse, or to use any badge or uniform appointed by the Council as the badge or uniform of a registered nurse, or of a registered male nurse, or of a registered mental nurse, or any colourable imitation of such badge or uniform, unless that person is registered as such under this Act, and, if any person knowingly takes or uses any such name, title, addition, or description or uses such badge or uniform or imitation thereof in contravention of this section, he or she shall be liable on summary conviction to a fine not exceeding ten pounds.

12. Copy of Register to be Evidence.—A copy of the Register certified to be a true copy by the secretary of the College of Nursing shall be evidence in all Courts of Law that nurses whose names are therein specified are registered under this Act, and the absence of the name of any nurse from the Register shall be evidence that such nurse is not registered under this Act;

Provided always, that in the case of any nurse whose name does not appear in the Register a certificate under the hand of the secretary that the name of such nurse has been entered on the Register shall be evidence that such nurse has been duly registered under this Act.

13. Penalty for Obtaining Certificate by False Representation and for Falsification of Register.—Any person—(1) who procures or attempts to procure a certificate under this Act by making or causing to be made or produced any false and fraudulent declaration, certificate, or representation, either in writing or otherwise; or (2) who wilfully makes or causes to be made any falsification in any manner relating to the Register, shall be guilty of a misdemeanour, and shall on conviction thereof be liable to be imprisoned, with or without hard labour, for any term not exceeding twelve months.

14. Provision as to Prosecutions.—A prosecution for any of the offences in this Act mentioned shall not be instituted by a private person without the consent of the College of Nursing, but such prosecution may be instituted by the College of Nursing.

15. Appeal from Decision of Council.—Any registered nurse, or registered male nurse, or registered mental nurse, aggrieved by a decision of the Council removing his or her name from the register may, within three months from the notification of such decision, appeal therefrom to the High Court of Justice in England and Wales, or to the Lord Ordinary, officiating on the Bills in the Court of Session in Scotland, or to the High Court of Justice in Ireland, and such appeal shall be final.

16. No Authority to Practise Medicine.—Nothing contained in this Act or in any rules made thereunder shall confer any authority to practise medicine, or to undertake the treatment or cure of disease.

17. Laying of Rules Before Parliament.—Rules under this Act shall be laid before both Houses of Parliament as soon as may be after they have been approved by the Privy Council.

